# Sound Symbolism Facilitates Word Learning in 14-Month-Olds

**DOI:** 10.1371/journal.pone.0116494

**Published:** 2015-02-19

**Authors:** Mutsumi Imai, Michiko Miyazaki, H. Henny Yeung, Shohei Hidaka, Katerina Kantartzis, Hiroyuki Okada, Sotaro Kita

**Affiliations:** 1 Department of Environment and Information Studies, Keio University, 5322 Endo, Fujisawa, Kanagawa, 252–8520, Japan; 2 School of Social Information Studies, Otsuma Women’s University, 2–7–1, Karakida, Tama-city, Tokyo, 206–8540, Japan; 3 Centre National de la Recherche Scientifique, Paris, France & Laboratoire Psychologie de la Perception, Université Paris Descartes, Paris Sorbonne Cité, Paris, France; 4 School of Knowledge Science, Japan Advanced Institute of Science and Technology, 1–1 Asahidai, Nomi, Ishikawa, 923–1292, Japan; 5 School of Psychology, University of Birmingham, B15 2TT, Birmingham, United Kingdom; 6 Brain Science Institute, Tamagawa University, 6–1–1, Tamagawa-gakuen, Machida-city, Tokyo, 194–8610, Japan; 7 Department of Psychology, University of Warwick, Coventry, United Kingdom; Goldsmiths, University of London, UNITED KINGDOM

## Abstract

Sound symbolism, or the nonarbitrary link between linguistic sound and meaning, has often been discussed in connection with language evolution, where the oral imitation of external events links phonetic forms with their referents (e.g., Ramachandran & Hubbard, 2001). In this research, we explore whether sound symbolism may also facilitate synchronic language learning in human infants. Sound symbolism may be a useful cue particularly at the earliest developmental stages of word learning, because it potentially provides a way of bootstrapping word meaning from perceptual information. Using an associative word learning paradigm, we demonstrated that 14-month-old infants could detect Köhler-type (1947) shape-sound symbolism, and could use this sensitivity in their effort to establish a word-referent association.

## Introduction

Traditional linguistics has long assumed that links between a word’s form and meaning are arbitrary [1]. However, words whose forms are motivated by their meanings (i.e., sound symbolic words) are commonly found across many languages of the world. For example, *bump* and *thump* sound like what they mean: an event with an abrupt end [2]. On a larger scale, phonological forms are often correlated with certain semantic classes (i.e., names of objects versus actions) [3]. Several languages even have large grammatically defined lexical classes of sound symbolic words (i.e., “ideophones,” “expressives,” or “mimetics”) [4,5]. A classic example of sound symbolism is the association between rounded vs. angular shapes and labels [6,7]. Presented with a forced choice, adults and children from different languages (e.g., German, English, and Swahili) much prefer to label rounded objects *maluma* and angular objects *takete* [8,9].

Sound symbolism has often been discussed in connection with language evolution, where oral imitation of external events links phonetic forms to their referents [7] [10,11]. Sound symbolic words may thus be "fossils" from earlier stages of language evolution, when sound symbolic links facilitated the rapid development of a common lexicon in human protolanguages [12,13]. This further suggests that sound symbolism may still facilitate synchronic language learning in infants and children.

Consider the classical induction problem in identifying intended word meanings [14,15]. Even when learners are explicitly given a novel label for an object that they can see [16], developmental research has shown that toddlers must recruit constellations of additional cues—conceptual, pragmatic and distributional (including shape or mutual exclusivity biases)—to infer word meanings in such situations [15,17,18,19,20]. Recent work has further shown that sound symbolism can similarly provide an inferential cue to word meanings [21,22], and even helps toddlers learn the meanings of novel verbs, which are generally more difficult than object names [23,24].

Many of the cues constraining word meanings listed above must be acquired through experience [25]. For infants just starting to learn words, however, the induction problem is harder [20,26], and these novice word learners are likely rely more on perceptual regularities over cognitive heuristics, as young infants can be constrained by limited information processing abilities [27]. Caregivers often create such regularities, for example, by synchronizing object movement and labeling [28], but it is not known whether infants also have more intrinsic perceptual biases for word learning (that could either come from inherent properties of the perceptual system, or from perceptual experience unrelated to language input). The present study asks whether sound symbolic links provide such a cue.

Evidence for sensitivity to sound symbolism in young infants is sparse, but recent research has shown that even 4-month-olds are sensitive to sound symbolism between size and vowels (i.e., [a] & [o] are mapped to larger things than [i] & [e], which are mapped to smaller things [29]). Ozturk, Krehm and Vouloumanos further reported that four-month-olds detected Köhler-type shape-sound symbolism [30], looking longer when a round shape was accompanied by the word “kiki” than it was associated with “bubu.”

In a recent study, using three EEG-based measures of brain activity, Asano et al.[31] showed that preverbal infants are sensitive to sound symbolism. In that study,11-mont-old infants were presented with a visual stimulus (e.g., a round shape) followed by a novel spoken word that either sound-symbolically matched ("moma") or mismatched ("kipi") the shape. Amplitude increase in the gamma band showed perceptual integration of visual and auditory stimuli in the match condition within 300 milliseconds of word onset. Furthermore, phase synchronization between electrodes at around 400 milliseconds revealed intensified large-scale, left-hemispheric communication between brain regions in the mismatch condition as compared to the match condition, indicating heightened processing effort when integration was more demanding. Finally, event-related brain potentials showed an increased adult-like N400 response—an index of semantic integration difficulty—in the mismatch as compared to the match condition. These findings suggest that 11-month-old infants spontaneously map auditory language onto visual experience by recruiting a cross-modal perceptual processing system and a nascent semantic network.

However, it is not known whether infants would use sound symbolism in a word learning context, especially for establishing an association between novel words and their referents. It could be that infants detect sound-shape correspondence [7,22] but this sensitivity is not used for word learning.

The present study investigated whether 14 month-old infants could detect Köhler-type shape-sound symbolism, and utilize this sensitivity to establish association between a word and a referent object. Here we chose infants at 14 months of age, who are just old enough to learn new words in laboratory tasks, but whose learning abilities are still very fragile, especially when trying to process the precise phonological forms of words [32,33]. We hypothesized that 14-month-olds detect sound-shape correspondence, given previous results with younger infants [29,30], and that this ability helps these infants make mappings from word forms to their referents. Specifically, we taught Japanese-learning infants two word labels, and then tested whether they encoded these labels in a preferential looking procedure. Half the infants were taught two sound symbolic labels (as rated by adults); half were taught the mismatching labels. We predicted that those in the sound symbolically matching condition would learn labels more easily than those in the mismatching condition.

## Method

### Participants

Participants were thirty-four full-term, monolingual Japanese 14-month-olds (*M* = 14 months; 16 days, range 13;27–15;09, 22 males). Infants were randomly assigned to either the match or mismatch condition (match: *n* = 17, *M = 14*:17, 11 males; mismatch: *n* = 17, *M* = 14:19, 11 males). An additional 11 infants were excluded from data analyses due to experimental error (*n* = 1), or fussiness (i.e., inability to complete the experiment due to crying or movement out of range of the camera [see Procedure], *n* = 10). All participants were recruited from the participant pool of Tamagawa University (Tokyo, Japan) via telephone calls or email. All adults and parents of infants gave written informed consent before participating in the study. This study was approved by the Ethics Committee of Tamagawa University.

### Apparatus

A black cloth curtain surrounded a 17-inch display (272 mm x 340 mm) where visual stimuli appeared, and a digital video camera was hidden below the screen, relaying video of the infant to the control area. Video was also recorded (29.97 fps) for offline coding of looking.

### Stimulus Matelials

Stimuli were constructed on the basis of a pilot study, where consonants (m, l, n, p, k), and vowels (a, o, i) that are related to smooth and jagged shapes—according to previous research on sound symbolism [6,22,34]—were rated. Seventeen bi-syllabic words were constructed by systematically combining these consonants and vowels (e.g., *kipi*, *kiki*, *pipu*, *mano*, *moma*, *noma*, *nono*, etc.), and eliminating words that were phonotactically illegal in Japanese, or that already had other meanings. Adult Arabic (*n* = 18), Japanese (*n* = 98) and English (*n* = 83) speakers were asked to choose the candidate word that best matched several variations of smooth/round and spiky/jagged shapes. The words *moma* and *kipi* were selected because their average rating (averaged across the three languages) was highest for the smooth/round and spiky/jagged shapes, respectively (see S1 Appendix).

**Fig 1:**
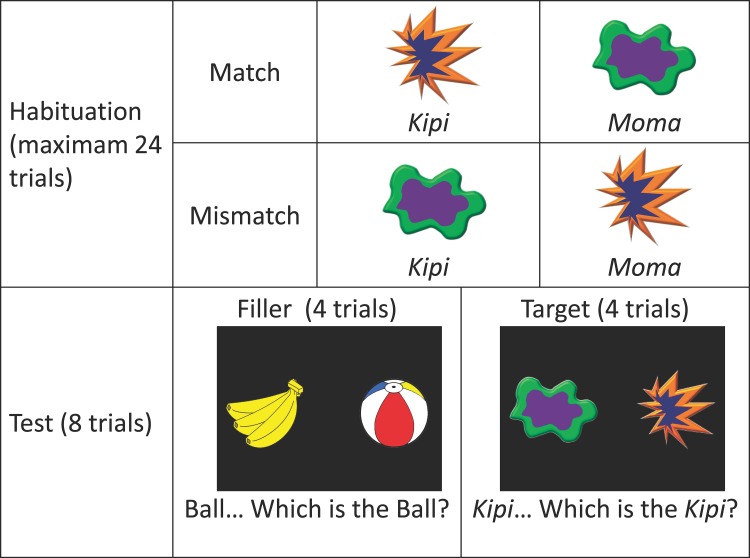
Design and Stimuli.

Target stimuli consisted of shapes that were composites of the shapes used during pilot testing. One was round, and one was spiky, and we also recorded audio tokens of the two novel words, *kipi* and *moma* (Fig. 1). Color was also added to the shapes to make them more interesting to infants. Selected colors were neutral with respect to the sound symbolism dimensions, and this was verified by ratings with additional English (*n* = 15) and Japanese (*n* = 20) speakers in a similar manner as the pilot study.

During a habituation phase, infants were presented with two pairs of audio-visual stimuli. Each object was presented in a 10 cm x 10 cm frame: in the match condition, *kipi*—spiky object and *moma*—round object; in the mismatch condition, *kipi*—round object, *moma*—spiky object. In a test phase, recognition of the target nonsense objects was tested along with several filler stimuli, consisting of two-dimensional color drawings of a ball, a banana, a car, and a picture book, each of which presented in the same size as the target objects and paired with audio recordings of the corresponding word in Japanese. These items were chosen based on normative data from the Japanese MacArthur-Bates Communicative Development Inventory (J-MCDI) [35]. A female Japanese speaker recorded the target and filler words, along with all other speech used in the experiment, in an infant-directed speech register.

### Procedure

Infants were tested individually in a quiet room on their parent's lap, positioned 60 cm from the display. Pretest, habituation, and test phases were presented, each of which contained several trials separated by a short attention-getting movie. Parents were instructed to keep their eyes closed during the experiment. After the completion of the experiment, they were asked to complete the J-MCDI.

**Pretest Phase.** Here we presented 4 familiarization and 2 referential trials in random order. Familiarization trials were meant to introduce infants to the objects used in subsequent phases, and the experiment involved presentation of side-by-side displays of either filler (2 trials) or target objects (2 trials). Each trial lasted 8 s and was accompanied by, "Mite! Mite!" (*Look!* *Look!*). Two referential trials were included to enhance infants' understanding of the referential nature of the labels [36], and here a single familiar object slowly moved (either up and down, or right and left) on the display, accompanied by the corresponding label in isolation and then in a carrier sentence (e.g., “Kuruma! Kuruma wo mite!” [*Car!* *Look at the car!*]).

**Habituation Phase.** The habituation phase consisted of a pseudo-randomly ordered series of trials such that each of the nonsense word-object pairings appeared twice in every block of four trials. In each trial, a single target object moved slowly from right to left in order to maintain infants’ attention [33], while one of the target words was paired with it (Fig. 1). Trials lasted a maximum of 16 s and were accompanied by 13 tokens of a target word, each spoken with a different intonation pattern. The habituation criterion was set to a maximum of 24 trials, or a decrease of 65% in looking to the longest previous block of 4 trials [32,37].

**Test Phase.** The test phase consisted of four filler and four target trials that contained two objects side-by-side, counterbalanced for side over the testing phase. The distance between stimuli was approximately 10 cm (10 deg). Two filler trials were always shown first, providing infants the opportunity to see familiar objects in the test procedure. The third and fourth trials were target trials, and the remaining four trials alternated between filler (F) and target (T) trials. All trials lasted 8 s, and began with a 3000 ms silent baseline period to measure visual preferences for the objects, followed by a phrase asking infants to look at the correct object (i.e., “X! X wa docchi?” [*X!* *Which is the X?*], where X stands for a filler or target word).

### Cording

Two blind coders classified each video frame as a look to the right, to the left, an ambiguous look, or no look. However, ambiguous looks were rarely observed and the wider consensus among the coders was that it was very hard to distinguish from No-look. Thus, in all analyses, we combined the data that were coded as “ambiguous” and a “no-look.” The data of 6 infants (18% of the sample) were coded twice to obtain inter-coder reliability, which was was high,κ = .83.

### Data Rejection

Individual trials were excluded if infants looked away more than 50% of the time (i.e. low attention [38]), or if infants showed 100% preference to a particular target in a trial (i.e. showing a “side-bias”, [39]). Seventeen of 136 filler trials (12.5%) and 41 of 136 target trials (30.1%) were excluded due to these criteria, which correspond with rejection rates in previous reports testing infants around this age in similar paradigms [37, 38]. One infant in the match condition did not contribute any data from target object trials after this criterion was applied, and so the analysis of the filler object trials includes all 34 infants tested, but the analysis for target object trials includes only 33 infants. It should be noted an analysis without these data rejection criteria resulted in a similar pattern of results (i.e., showing the critical interaction between looking in match/mismatch condition, and pre-/post-naming phases, see below), but these criteria were still applied so as to ensure that data came from infants who were both on-task (i.e., the low attention criterion), and had seen both objects (i.e., the side-bias criterion).

In addition, overall participant attrition rates (24% of the babies tested were rejected, mostly due to fussiness) were high, but this rate is similar to other studies that have used similar paradigms to test infants from 14 to 18 months of age (49% in [36], 35% in [37], 23% in [38]).

## Results

### Vocabulary analysis

The J-MCDI data were obtained from 28 out of 34 parents of infants by mail within one month after the infants’ participation of the experiment. Comprehension on the J-MCDI ranged from 9 to 287 words (*Mdn* = 51, *M* = 72.3, *SD* = 68.1) and reported production ranged from 0 to 73 words (*Mdn* = 3, *M* = 9.3, *SD* = 16.1). Comprehension of the filler words was at 50.9%, meaning that, on average, infants knew only 2 out of four filler words. Thus, although the norms on the J-MCDI suggested that a majority of 14-month-old Japanese infants should know the words used for the filler trials, these words were not so familiar to the infants in our sample.

Moreover, infants in match and mismatch conditions did not differ on their vocabulary size in either comprehension or expression, and did not differ on the reported number of known filler words (the Mann-Whitney U test was used, since MCDI scores were non-normally distributed: comprehension, *U* = 79.5, *p* = .40; expression, *U* = 95.5, *p* = .91; knowledge of filler words, *U* = 98.0, *p* = .99).

### Pretest and Habituation Trials

To ensure that infants in both match and mismatch conditions had equal exposure to the target objects before moving on to the subsequent phases of the experiment, we calculated the time that infants in match and mismatch conditions looked to the two trials containing the target objects during these preliminary phases. No differences were observed, *t* (29) = 1.08, *p* = .29.

The mean number of trials required to reach habituation was 10.3 for the Match condition (*SD* = 3.7, range: 6–16) and 9.6 for the Mismatch condition (*SD* = 4.9, range: 6–23), which did not differ across the two conditions (*t* (32) = .52, *p* = .61). All infants successfully habituated.

### Test Trials

**Fig 2:**
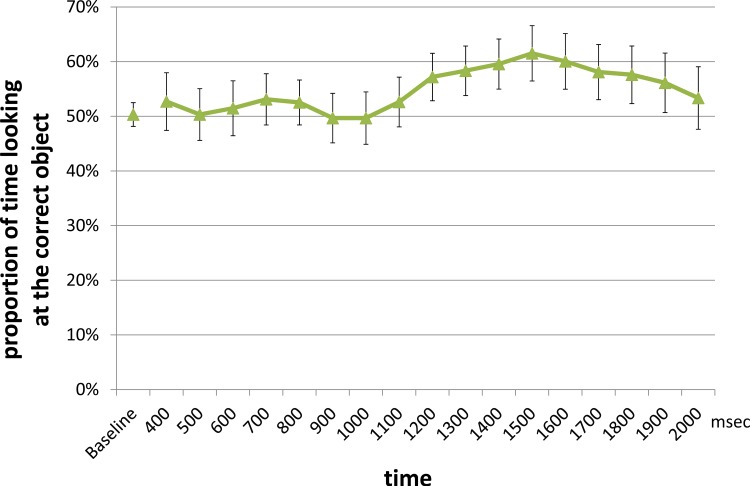
Mean proportion of time looking at the correct object in 100 ms intervals. In the filler trials match-mismatch combined. Baseline looking was calculated by averaging infants’ looking at the target object 3000 ms immediately prior to the word onset. The error bar indicates standard error.

**Filler Objects: Time Course Analysis.** Because we did not predict differences across filler trials for infants in the match and mismatch conditions, we aggregated the data across the two conditions for this analysis. In previous research assessing 14- and 15-month-old infants’ ability to identify referents of familiar words, it is estimated that infants begin to fixate the correct object from approximately 367 ms, until 1800 ms—2500 ms after the onset of the first instance of the target word [37,39,40,41]. Here we chose a similar dependent measure, as well a similar time window for analysis as used in [36] and [40]. The mean proportion of the looking to the correct object (relative to the looking time to both objects) was calculated across all infants during a 400 ms—2000 ms time window after the onset of the target word, and was then compared against the mean proportion of looking to the same object during the baseline period (i.e., the 3000 ms period immediately preceding the word onset during which the infants saw the two shapes side by side without hearing the sound). These proportions in the target time window are plotted in 100 ms bins in Fig. 2, as we do not know in advance how the time course of infants’ looking behavior would be.

Results revealed that the proportion of looking at the correct object during the target time window was not significantly different from that during the baseline time window (baseline, *M* = 50.3%; target window, *M* = 54.7%; *t* (33) = 1.55, *p* = 0.13, although visual inspection of the data suggest that a more restrictive time window may have revealed more robust results. However, without any *a priori* motivation for changing the time window of analysis, our sample of Japanese 14-month-olds did not clearly show any evidence of looking to the correct object when hearing the words, *ball*, *banana*, *car*, or *book*.

**Fig 3:**
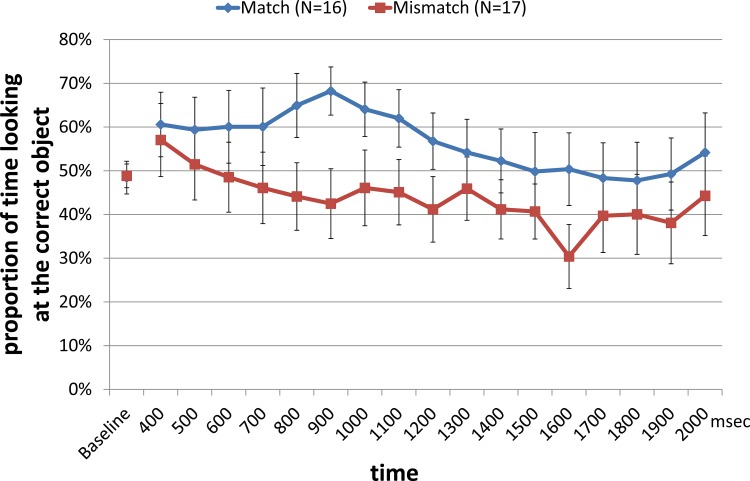
Mean proportion of time looking at the correct target object in 100 ms intervals in the test trials. Baseline looking was calculated by averaging infants’ looking at the target object 3000 ms immediately prior to the word onset. The error bar indicates standard error.

**Target Objects: Time Course Analysis.** As was done for the filler objects, the mean proportion of infants’ looking to the correct object relative to both objects (i.e., the correct object was the one associated with the novel word during the habituation phase) was calculated for each 100 ms starting from 400 ms—2000 ms from the target word onset, and was plotted separately for the match and mismatch condition in Fig. 3.

**Fig 4:**
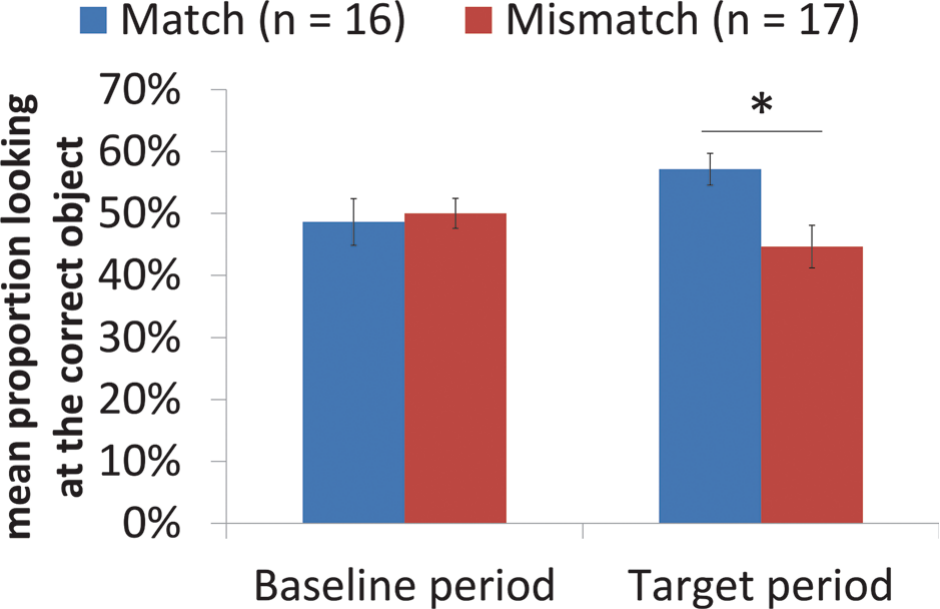
Mean proportion of time looking at the correct object in each time windows in the test trials. Calculation of baseline looking was the same as Fig. 3. Looking of Target window was calculated by averaging infants’ looking at the target object from 400 ms to 2000 ms. The error bar indicates standard error.

The same time window analysis as was performed with the filler trials was conducted here on test trials. A 2 (baseline vs. target time window) X 2 (match vs. mismatch) ANOVA analyses was conducted on mean proportion looking at the correct object. As in the filler trials, the target time window was 400 ms—2000 ms after the onset of the word, and baseline looking was the 3000 ms time window immediately prior to the word onset (Fig. 4).

Consistent with the sound-symbolism bootstrapping hypothesis, the interaction between the factors of Time Window and the Condition is significant, *F* (1, 31) = 4.31, *p* = .046, *η* ^*2*^ = .11. In order to tease apart this interaction, two follow-up multiple testing of simple main effect were conducted within each time window. The first analysis showed that there were no differences between infants in the match (*M* = 48.6% looking to the correct object, *SD* = 14.6%) and mismatch conditions (*M* = 50.0% looking to the correct object, *SD* = 9.7%) during the baseline, *F*(1, 31) = .11, *p* = .75, *η* ^*2*^ = .003. The second analysis showed that infants in the match condition (57.1% looking to the correct object, *SD* = 10.0%) looked significantly longer to the correct target object than infants in the mismatch condition (*M* = 44.6% looking to the correct object, *SD* = 13.8%) *F* (1, 31) = 8.83, *p* <. 025 (bonferonni corrected), *η* ^*2*^ = .222. Together, these results suggest that there were no differences between match and mismatch conditions in the baseline period, but that match babies outperformed mismatch babies in the critical post-naming time window.

An alternative way of analyzing this interaction is to examine changes across time windows within each group of infants, using the same analysis as above. Within the match condition, proportion looking to the correct object marginally increased across time windows, *F*(*1*, 31) = 3.25, *p* = .081, η^2^ = .095. However, in the mismatch condition, proportion looking to the correct object across time windows did not change, *F*(*1*, 31) = 1.29, *p* = .265, η^2^ = .040. These results again suggest that infants in the match and mismatch conditions had different patterns of looking.

The results of the ANOVA analysis were certainly *consistent with* what was expected by the hypothesis. However, when a qualitative view of the data as seen in Fig. 3 is considered, visual inspection of the data suggests that the time course of looking to the target object was different when comparing across the match and mismatch conditions. In the match condition, infants seemed to look towards the correct object in the first part of the time window (from around 800 ms—1000 ms after words onset). However, after that, their attention gradually decreases toward chance level (50%). In the mismatch condition, infants appeared to look at both objects equally in the first part of the time window, but their looking to the correct object continuously declined throughout the time course, staying around just below 50%.

These visual inspections suggest that infants in the match condition showed early recognition of the named object, but that infants in the mismatch did not, instead gradually showing a trend to look at the other, non-trained object, which was sound symbolically matching to the sound. The ANOVA analysis over the whole time window (400 ms—2000 ms), above, ignores these differences in time course. To further explore our data, we turned to a Bayesian analysis of looking times in order to better assess the contributions of infants’ dynamic looking patterns to our experimental question.

**Target Objects: Bayesian Analysis.** The above time window analysis suggests that infants in the match condition outperformed those in the mismatch condition, looking more at the trained object when hearing a target word. However, an analysis with traditional linear models such as ANOVA pose some limitations for our data set, in which infants’ looking data shifts dynamically along the time course in different patterns, which may also differ across the conditions to be compared. Thus, we use a Bayesian analysis to address several shortcomings of the time window analysis above.

First, the time window analysis ignores dynamic looking patterns, as the implicit idea in that analysis is that infants’ looking to the target object does not vary in interesting ways within the pre-determined time window. However, Fig. 3 clearly shows that this assumption does not hold in our case. This compounds the more general concern about the appropriateness of our 400 ms—2000 ms time window. Indeed, we had chosen this time window based on previous work examining 14-month-old infants’ word recognition and learning abilities in similar paradigms [37,41], but there is wide disagreement about the appropriate location of time window onsets and offsets when analyzing word learning and recognition from 12 to 15 months of age [39,40,42,43,44].

Second, it is not yet clear whether the difference between the match and mismatch conditions can be truly attributed to the effect of sound symbolism on training. For example, the difference between the two conditions may simply reflect a global preference to look at the object that sound symbolically matched the label, irrespective of the training condition. (Note that in the mismatch condition, the [incorrect] distractor item in the test trials is also the sound symbolically matching word). Nevertheless, this alternative interpretation seems unlikely given the data in Fig. 3: The time course of looking to the sound symbolically matching object (i.e., the trained object for the match condition, and the non-trained object in the mismatch condition) should be symmetrical if the same mechanism were driving looking in both conditions. The patterns observed in Fig. 3 indicate otherwise, but the time window analysis used in previous work assessing preferential looking in infants cannot statistically separate these two interpretations.

To circumvent the problems described above, we conducted a Bayesian analysis similar to the one proposed by Yurovsky et al. [45]. Their analysis has at least three advantages over traditional linear models for looking-time data. First, looking behavior is analyzed as a continuous time series. This means that, instead of averaging the looking time over a pre-determined time-window, all the frame-by-frame data points are used for the analysis. The analysis specifies an optimal “time window” for a given experimental setting through parameter estimation of the dataset. Second, instead of assuming a linear increase or decrease of looking time, this analysis considers the probability of looking as a polynomial function of time with multiple factors as its coefficients. With nonlinear modeling of looking behaviors, the effects of the experimental manipulation can be separated from other subsidiary factors such as infants’ response biases. Third, traditional linear models simply treat individual difference in infants’ looking profiles (e.g., a preference to look at a certain location on the monitor, a preference to look at a particular object, the likelihood to shift eye-gaze, etc.) as experimental noise, which weakens statistical power to detect the experimental effect of interest. As a result, the critical experimental effect may be washed out. In contrast, Yurovsky et al.’s Bayesian model is able to consider fine-grained response characteristics for a particular subgroup of infants by classifying participants with similar looking patterns into clusters and estimating group parameters.

In analyzing our data, hypothetical relationships among a set of experimental factors (i.e., effects due to experimental manipulations: training, sound symbolism at test, and their interaction), subsidiary factors (i.e., a bias to look toward an specific object or a specific location in the monitor), and potential patterns of behaviors (i.e., looking time) are expressed as a statistical model with a set of parameters, which is estimated through model fitting to a dataset. The model consists of a set of parameters, which also include preference parameters for factors that could potentially affect the looking of a particular area of interest (AOI) at a given moment: individual infants’ preference to look at a particular location (i.e., left or right or neither), a preference to look at a particular object, a preference to look at the “correct” object with which the label was associated, or a preference to look at the object that was sound symbolically matching. These parameters were estimated with respect to the subjects’ individual variances using a hierarchical Bayes model (see S2 Appendix for the detail).

Looking data from each of the video frames from all target trials were classified as a look to the left AOI, to the right AOI, or ‘No-look’. As this model incorporated information about no looking, as well as infants’ preference for a particular object, the elimination criteria from the ANOVA analyses (section 2.6 above) no longer applied. Instead, frame-by-frame counts for the entire trial were analyzed as a function of five factors: a location-specific preference, an object-specific preference, a “correct” (trained) object preference, a sound symbolism preference, and most importantly, the interaction between training and the sound symbolic match, which indicates the bootstrapping effect of sound symbolism for training.

The location-specific preference is defined as a bias to look at a particular side of the screen. The object preference is a bias to look toward a particular object. The remaining three factors are factors directly relevant to experimental manipulations. The correct object preference is a preference to look at the object with which the label was associated during the habituation phase. The sound symbolism preference is a tendency to look at the sound symbolically matching object (to the label heard in the trial) after the onset of the speech (i.e., the preference for the spiky object after the onset of speech “kipi,” or the preference for the round object after “moma”). Finally, the interaction effect is a joint effect of the training and sound symbolism that cannot be explained by the effect of sound symbolism alone or the effect of training alone, which was the critical effect of interest in this analysis.

### Results of the Bayesian analysis

We modeled the probability of looking at an object (after the onset of the target word) as a function of its various properties: the three experimental factors (training, sound-symbolic match at test, and the training-sound-symbolism interaction), whose influence was assumed to change as a function of time within a trial, two subsidiary preference factors (object shape and object location), assumed to be constant over time. We evaluated whether the model should include all three experimental factors and what type of time-dependency of the three experimental factors (a linear, quadratic, or cubic function of time) was optimal. To compare these possible models, we ran a series of (Bayesian) models that examined experiment-related factors (see Table 1).

**Table 1:** Summary of the Bayesian hypotheses testing.

Models					Results
Models	Tr	SS	Tr-SS	Poly	Log-BF of P2-full to respective models
P1-full	Yes	Yes	Yes	1	27.584
P2-full*	Yes	Yes	Yes	2	0
P3-full	Yes	Yes	Yes	3	247.378
P2-NoInteraction	Yes	Yes	-	2	109.625
P2-NoSS	Yes	-	Yes	2	119.609
P2-NoTraining	-	Yes	Yes	2	92.546

(BF = Bayesian factor, Tr = the factor for training, SS = the factor for sound symbolic match at test, Tr-SS = the interaction between Tr and SS, Poly = the order of polynominal function).

In Bayesian hypothesis testing, each model specifies a probability for the hypothesis to reproduce the current dataset. Through the process of model fitting, we estimated a set of parameters for all of the five factors described above (i.e., three experimental factors and two factors reflecting individual looking biases), but we report only the experiment-relevant factors here (i.e., training, sound-symbolic match at test, and training-sound-symbolism interaction factors).

The effect of each factor is evaluated by a *Bayes factor* ([46] See also [47] for a review in psychological studies). According to Jeffreys (1961), “[t]he Bayes factor (BF) *X* of Model *A* to *B* given a dataset indicates that the odds ratio for Model *A* to reproduce the dataset is *X* times higher than that for Model *B* to do so under an even prior probability for each model.” Here we use a derivative of the BF, the log-BFβ, which indicates that the probability of reproducing the data is exp(β) times higher as compared to the base model. A BF from 3 to 10 (log-BF from 1.1 to 2.3) indicates a “substantial” evidence for the hypothesis, a BF from 10 to 30 (log-BF from 2.3 to 3.4) indicates “strong” evidence, and a BF above 30 (a log-BF greater than 3.4) indicates “very strong” evidence [46]. In the present study, we considered log-BF values larger than 3.4 to be a strong indication that Model A is preferred over Model B, following Jeffreys’ (1961) criterion.

Table 1 shows the results of our model fitting procedure (In a preliminary analysis, we analyzed 16 possible models, crossing the level of complexity (P1, P2, P3, and P4) with model type (full, NoSS, NoTr, and NoInt). Again, we found here that the P2-full model had the best fit, but only 6 of these models are shown in Table 1 due to the assumptions behind the sparseness prior function (see text), and for reasons of rhetorical simplicity). The first three models—P1-full, P2-full, and P3-full models—were full models in which all of the three experimental factors were included. These three models assumed different levels of complexity in their functions of looking probability. The P1-full model assumes a first order polynomial function, and the P2-full and P3-full models assume a second- and third-order polynomial function, respectively. We compared the three models to determine the optimal polynomial function. The analysis of log-BF indicates that the second-order polynomial function (i.e., the middle degree of complexity: P2-full) is strongly favored over the relatively simple model (P2-full to P1-full: log-BF 27.584), or over the complex model (P2-full to P3-full: log-BF 247.378). We therefore employed the P2-full model as the baseline model, against which each of the subset models was compared.

Because the current model utilizes a “sparseness prior function,” which adaptively removes unnecessary parameters that do not contribute to data reconstruction, the current model is biased toward simpler models. Thus, it is unlikely that redundant models with unnecessary parameters are erroneously chosen. This is why we did not include models that are simpler than the listed models shown in Table 1 and believe that the tested models listed Table 1 cover all possible models that should be considered. Moreover, P4 or higher models are also unlikely when the P3 models (n = 3) are evaluated to be poorer than those of the P2 models. We thus do not report P4 or more complex models as they are even more redundant than the P3 models.

In the P2-NoSS (No Sound Symbolism) model, the sound symbolism factor is removed, and was compared to the P2-full model. Likewise, the P2-NoTr (No Training) model excludes the training factor from the full model, and a comparison between the P2-full model and the P2-NoTr (No training) allowed us to estimate the effect of training alone. The P2-NoInt model includes the sound symbolism factor and the training factor, but the interaction was excluded. This model was the most critical one, as here we can estimate whether sound symbolism enhanced training over and above the effects of training alone and/or sound symbolism alone.

The analysis of the Bayes factors suggested that the P2-full model was strongly favored over the P2-NoSS (logBF 119.609), P2-NoTr (log-BF 92.546), and P2-NoInt (log-BF 109.625) models.These results thus indicate that the effects of sound symbolism, training, and the interaction between sound symbolism and training all significantly contributed to the model fit. In other words, infants tended to look at the trained object regardless of whether this object was sound symbolically matching or not. Moreover, sound symbolism affected infants’ looking, regardless of whether infants were trained on a sound symbolically matching object. Finally, the Bayesian analysis also established that these factors interact. In conjunction with Fig. 3, it suggests that sound symbolism provides additional boost to the training especially in the first 800ms such that the training effect was stronger for the infants in the match condition than those in the mismatch condition. The details of the estimated effects of training, sound symbolism, and interaction are shown in S3 Appendix.

## Discussion

This research examined whether sound symbolism helps word learning in 14 month-old infants. The results indicated that infants looked at the correct referent object (trained during the habituation period) more often, especially in the first 800 ms after the word onset, when the word and the object sound symbolically matched. This suggests that sound symbolism boosted learning of form-meaning mappings.

Around this age, word learning mainly involves establishing association between words and referents, and this process is still effortful [32,33,48] as infants have limited information processing capacities and little experience in mapping words to the world. They may rely more on perceptually based cross-modal correspondences between speech and visual input in word mapping [20,27] and previous work suggested that sound symbolism is one such cross-modal correspondence that may be available to infants [29,30]. Our results tested this hypothesis in infants just beginning to learn words, and results suggest that 14-month-old infants use sound-symbolic correspondences between speech sounds and object properties as just such a mapping strategy.

It is possible that the sensitivity to sound symbolism in Japanese infants is influenced by hearing sound symbolic words more frequently in daily basis than infants who are acquiring languages like English [49,50]. The current data of course do not exclude this possibility. However, as we have reviewed, previous studies showed that infants reared in a language who do not have a productive system of sound symbolic lexicon are sensitive to sound symbolism [29]. Furthermore, we have demonstrated that 3-year-old English-speaking were able to learn verbs better when novel verbs were created from Japanese mimetics (doing nosu-nosu) as compared to English-sounding nonsense words (fepping). If children as old as three benefitted from sound symbolism, it is likely that infants who have much less experience in word learning can also benefit from sound symbolism regardless of the language environment they are reared in.

It should also be noted that the Japanese infants we investigated did not show evidence that they understood familiar words used for the filler trials. We selected the words (ball, car, picture book, balloon) based on the norming data reported in the J-MCDI. However, when we asked the mothers after the completion of the experiment, only half of the infants in our sample knew these words. The J-MCDI was published only a few years ago, and there is relatively little data in the literature about how norming data provided by the developer of the J-MCDI is related to word recognition abilities in experimental settings [51].

The fact that sound symbolism interacts with early word learning supports two distinct, but related hypotheses. First, sound symbolism may allow infants to anchor speech to meaning, which in turn helps them obtain “referential insight”—the insight that language sounds are symbols that represent concepts [27]. Second, sound symbolism may also help language learners understand how complex meanings are constructed in languages with sound symbolically productive lexical classes. These sound symbolic words may have common sound features across distinct words with common semantic components. For example, consider Japanese sound symbolic words like "goro" (a heavy object rolling), "guru" (a heavy object rotating), "koro" (a light object rolling), and "kuru" (a light object rotating). In this set, it is easy to extract a subpart of words (e.g., a combination of a velar consonant and /r/) with a consistent meaning (e.g., circular movements), which can help infants become aware of morphological decomposition. This could, in turn, further help a learner realize combinatory properties of language (i.e., that complex meanings are derived from combinations of simpler meanings), seen not only in words but also in syntactic patterns [52]. Once a learner has acquired sound-symbolically based systems relating surface structure to meaning, they may be able to use this early knowledge to bootstrap themselves to more abstract meanings, needing direct perceptual anchors less and less, perhaps reflecting similar trajectories in language evolution [24].

A recent report raises interesting issues that seem to be at odds with these conclusions, reporting instead that sound symbolism does not help the learning of one-to-one word-object mappings when (adult) learners acquire the labels of *similar* objects[53]. In that report, sound symbolism facilitated the association between distinct classes of referents (e.g., a set of pointy versus smooth shapes) with distinct classes of sounds (e.g., words with plosive versus continuant consonants), but at the cost of learning individual mappings between a single shape and a precise phonetic form (see also[54] for a related point about the *usefulness* of arbitrary sound-meaning correspondences for learning in ambiguous situations).

Sound symbolism may be helpful in learning many kinds of words, but the way in which it is helpful likely changes at different points in development. Many of infants’ first words are basic-level terms that refer to category prototypes (a robin is a *bird*, but a penguin is not), and thus young infants are not likely to face the kinds of situations that Monaghan et al. [48] had in mind, where similar phonological words have to be mapped onto similar shape categories. Sound symbolism may instead help infants identify the particular part of an ambiguous scene as the referent: When hearing *kipi*, infants may selectively attend to pointiness in visual shapes, which in some cases may help to guide them to the correct referent. Indeed, recent data by Monaghan, Shillcock, Christiansen, and Kirby [55] have suggested that the acquisition of English words follows just such a pattern: earlier acquired words have a tendency to be more sound symbolic than later acquired words.

As vocabularies grow, children—particularly those learning a language with productive lexicalized systems of sound symbolism—may find themselves in a situation more similar to the one described in [48], where similar words from the same class must be distinguished (e.g., the distinction between *goro* and *guru* in Japanese). Sound symbolism may not facilitate the learning of distinct individual mappings within a class, but by this age children likely learn words using more sophisticated (less perceptual) strategies. Indeed, the benefit that sound symbolism may have at this stage is likely to be qualitatively different, perhaps helping learn the combinatorial properties of language in a segregated, closed set of words.

Future work will benefit by replicating the Bayesian analysis techniques that we have used here, which revealed separable (and interactive) effects of sound symbolism and of form-meaning mappings in the time course of word recognition. Subsequent studies might compare, for example, sound-symbolic mapping situations to situations where sound symbolism plays no obvious role. Such work could independently isolate the effects of sound symbolism and of learned mappings between form and meaning, confirming the results we report here. We nevertheless present preliminary evidence for the idea that infants at the beginning stages of word learning can use sound symbolism to map words onto objects. This cue for word learning may provide an important insight into one of the greatest questions concerning the ontogenesis of language, namely, how infants who do not yet have much previous experience in word learning can initiate the mapping between the world and language symbols, because this cue is likely to be biologically endowed and available at the very first stage of word learning [22,29,30].

## Supporting Information

S1 Appendix The stimuli construction. (PDF)

S2 Appendix The Bayesian data analysis. (PDF)

S3 Appendix The Bayesian data analysis. (PDF)
