# Supplementary material for: Sound Symbolism Facilitates Word Learning in 14-Month-Olds
Source: PLoS One. 2015 Feb 19;10(2):e0116494. doi: 10.1371/journal.pone.0116494 (PMC4335030; doi:10.1371/journal.pone.0116494)
Supplement: S1 Appendix — (PDF) [file pone.0116494.s001.pdf]

Appendix S1: The stimuli construction

Adult Arabic (n = 18), Japanese (n = 98) and English (n = 83) speakers were asked to choose one of the 17 candidate words for each of six shapes, and Table S1 illustrates the average percentage that each word was selected as the best match across all three variations of smooth/round shapes, and three variations of spiky/jagged shapes. The words *moma* and *kipi* were selected because adults frequently chose these as the best match for the smooth/round and spiky/jagged shapes, respectively (*kiki* was only later excluded—although not a word in Japanese, it is the name of a popular movie character, and in English it is a possible first name).

Table S1. Average percent of the time that each word was chosen as the best match for rounded and spiky objects

	**Kipi**	Kiki	kipu	kupi	Kupu	Piki	Pipu	puki	**moma**	mamo	Mana	mano	Mona	namo	noma	nona	nono
Rounded objects																	
Japanese	0.51%	0.41%	0.71%	1.46%	4.75%	0.21%	4.14%	2.78%	9.16%	9.26%	7.10%	8.21%	7.60%	8.38%	10.52%	10.39%	14.40%
English	2.20%	0.97%	2.37%	1.58%	4.31%	1.14%	4.56%	2.28%	13.24%	13.08%	10.73%	6.34%	7.75%	6.78%	6.86%	6.84%	8.96%
Arabic	0.37%	0.96%	2.45%	3.19%	5.30%	1.69%	6.91%	5.64%	11.90%	8.63%	5.87%	8.25%	7.51%	10.66%	6.04%	6.94%	7.70%
Average	1.03%	0.78%	1.85%	2.08%	4.79%	1.01%	5.21%	3.57%	11.43%	10.32%	7.90%	7.60%	7.62%	8.61%	7.81%	8.06%	10.35%
Spiky objects																	
Japanese	23.39%	24.25%	10.34%	8.70%	5.13%	13.13%	3.74%	7.04%	0.37%	0.51%	0.48%	0.44%	0.27%	0.61%	0.44%	0.78%	0.37%
English	11.53%	14.59%	9.33%	7.30%	8.80%	13.02%	7.21%	9.50%	0.35%	0.97%	3.08%	2.19%	0.71%	4.31%	1.50%	2.55%	3.07%
Arabic	13.37%	19.15%	9.23%	8.35%	5.28%	13.62%	6.60%	12.31%	1.91%	0.38%	1.51%	0.95%	1.71%	2.05%	0.57%	1.68%	1.32%
Average	16.10%	19.33*%	9.63%	8.12%	6.40%	13.26%	5.85%	9.62%	0.88%	0.62%	1.69%	1.19%	0.90%	2.32%	0.83%	1.67%	1.59%

**kiki* was not chosen because this can be an existing proper name in English and Japanese.
