# Supplementary material for: Sound Symbolism Facilitates Word Learning in 14-Month-Olds
Source: PLoS One. 2015 Feb 19;10(2):e0116494. doi: 10.1371/journal.pone.0116494 (PMC4335030; doi:10.1371/journal.pone.0116494)
Supplement: S2 Appendix — (PDF) [file pone.0116494.s002.pdf]

Appendix S2: The Bayesian data analysis

In the analysis, we assume that the following set of parameters,

$$\Theta = \{\lambda_l, \lambda_i, \sigma_j, \theta_j, \bar{\theta}_j\}_{l \in \{L,R,\phi\}, i=1,\ldots,m, j=1,\ldots,n},$$

where λ_l is a location preference parameter for each of the three AOIs: left, right or no-look denoted by $\{L, R, \phi\}$ respectively; λ_i $(i = 1, 2)$ is a parameter for object preference for each of the 2 objects; $\{\sigma_j, \theta_j, \bar{\theta}_j\}$ $(j = 1, \ldots, n)$ represent parameters for the training, sound-symbolism, and training-and-sound-symbolism interaction, respectively, up to the n-th order of polynomial. This interaction parameter captures the specific effect that arises from seeing the object that was trained and symbolic-match object as compared to seeing the object that was trained but not sound symbolic or the object that was sound symbolic but not trained. Thus, this effect is not relevant to the infants in the sound symbolic mismatch group, as they did not experience the same objects in this context. For each participant, the probability of looking to AOI y at the moment τ of the trial t is proportional to the exponential of the combined factor X, $P(Look, y, t, \tau \mid \Theta) \propto \exp(X_{lt\tau})$. The time varies from 0 to 3 (from the onset of the speech to the end of the trial in second), and the time-dependent variables (e.g., training) are assumed to have no effect before the onset. Preference for AOI, either left (L) or right (R), is a linear combination of the factors: a location bias λ_l, an object bias λ_i, a time-dependent sound-symbolic-matching object preference σ_j and the habituated-object preference θ_j. We define $X_{lt\tau} = \lambda_l + \sum_{i=1}^{m} \lambda_i O_{ilt} + \sum_{j}^{k} \tau^j (\sigma_j S_{lt} + \theta_j H_{lt} + \bar{\theta} j S_{lt} H_{lt})$, where O_{iLt} is 1 if the object i is at the AOI l in the trial t and otherwise 0; S_{Lt} is 1 if the object at AOI l in trial t is sound-symbolic matched and otherwise 0; H_{lt} is 1 if the object at AOI l in trial t is habituated and otherwise 0. A special AOI ϕ, denoting looking-away from stimuli, has a constant 0 as baseline preference, $X_{\phi t\tau}$. For each participant, we denote the set of the binary dependent variables $D = \{D_{lt\tau}\}$, which indicates whether the participant looked (1) or did not look (0) at the AOI y for each moment τ of the trial t given the parameter set Θ. Then, the conditional probability of the looking D given the set of independent variables $\{D_{lt\tau}\}$ is

$$P(D \mid \Theta) = \prod_{t,\tau,l \in \{L,R,\phi\}} \left(\frac{\exp(X_{lt\tau})}{\sum_{t,\tau,l} \exp(X_{lt\tau})} \right)^{D_{lt\tau}}.$$

The prior distribution for each parameter of Θ was set to be a normal distribution with mean 0 and standard deviation σ, which follows the (Jeffreys' uninformative) gamma distribution. This prior setting means that the model will nullify the effects of unneeded parameters by default. This prior distribution is exactly the same as the one described in [44]. Thus, see the reference [44] for the details.
