# Supplementary material for: Sound Symbolism Facilitates Word Learning in 14-Month-Olds
Source: PLoS One. 2015 Feb 19;10(2):e0116494. doi: 10.1371/journal.pone.0116494 (PMC4335030; doi:10.1371/journal.pone.0116494)
Supplement: S3 Appendix — (PDF) [file pone.0116494.s003.pdf]

Appendix S3: The estimated parameters in the Bayesian data analysis

This appendix focuses on how the three experimental factors (the sound symbolic match at test, the trained object during habituation, and the interaction between the sound symbolic match at test, and the training) can model looking probability. As detailed in Appendix S2, looking probability is modelled as a second-order function containing a polynominal, $a\tau^2 + b\tau + c$, where τ is time from the stimulus onset, a is the second order coefficient, b is the first order coefficient, and c is the constant. The three experimental factors contribute to the first and second order coefficients. Because of the way we coded the interaction term (see Appendix S2), larger coefficients for the interaction term indicate an extra boost in looking probability when the object is trained during habituation (i.e., the correct referent object) and is sound symbolically matching. The factors of object preference and the location preference contribute to the constant. The Baysian analysis upon which we based our analysis divides participants into groups according to similarilty of looking behaviours [45], and Table S2 shows the estimated parameters for each of seven groups in the P2-full model. In the Table S2, each column indicates a parameter value, ID: the group ID, # match: the number of infants in the match condition withn the group, # mismatch: the number of infants in the mismatch condition within the group, Training, Sound symbolic match, and Interaction: median and 95% credible intervals of the posterior distribution of coefficients for the first and second order polynomial. The bolded parameters indicate a significant difference from zero, which is defined either by the 95%-lower bound larger than zero or by the 95%-upper bound smaller than zero. In the present study, the signifiant interaction between training and sound symbolic match at test is most important for our conclusion. In particular, it is important at what time point the objects that were trained during habituation *and* sound symbolically matched the speech at test lead to extra looking time. This is determined by the peak and the width of the polynominal function (a parabolla), representing the coeffient of the interaction term in the time course. These can, in turn, be determined by the first and second order coefficients. Due to coding conventions, a positive first order coefficient indicates an effect of this interaction early in the time course. A negative second order coefficient indicates that the effect changes across time, or that looking is in a convex-upward form (i.e., the effect goes up and then goes down). The timing of the peak depends on the combination of the two coefficients. For illustrative purposes, Figure S1 indicates the effect of this interaction term as a function of time for all infants. A look at Table S2 shows that Groups 1, 2, 3, and 5 showed significant effects of the interaction in either first or second order coefficient (28 out of 34 infants). For a majority of those infants (i.e., those classified in Groups 1, 2, and 3; 23 out of 28), sound symbolism gave an additional boost for the training effect at an early period of the time course, as the coeffient of the interaction term showed an early peak and was mostly positive early on (before 800 ms, according to Figure 3). For a small number of children in

Group 5 (5 out of 28), sound symbolism attenuated the training effect.

Table S 2. The estimated parameters for each of the seven groups of participants.

ID	#match[a]/ #mismatch	Training		Sound symbolic match at test		Interaction	
		1st order	2nd order	1st order	2nd order	1st order	2nd order
1	2/ 5	**-7.36**[b] **(-8.5, -5.4)**[c]	**3.10 (1.4, 4.1)**	**-4.90 (-5.7, -3.1)**	-0.02 (-1.9, 0.3)	**14.51 (10.8, 15.9)**	**-5.24 (-7.1, -2.2)**
2	3/ 2	**-2.77 (-3.5, -2.3)**	0.00 (-0.4, 0.6)	**-2.58 (-3.6, -1.8)**	1.52 (-0.04, 2.8)	**2.70 (1.6, 3.7)**	0.00 (-0.6, 1.3)
3	6/ 5	**-7.49 (-8.3, -6.4)**	**6.59 (5.4, 7.5)**	**-4.78 (-5.6, -4.1)**	**3.40 (2.6, 4.5)**	**10.99 (9.7, 12.4)**	**-8.25 (-10.0, -6.8)**
4	2/ 1	-0.00 (-0.3, 0.3)	-0.00 (-0.3, 0.3)	**3.39 (2.1, 4.4)**	**-6.89 (-8.90, -5.1)**	0.08 (-0.30, 1.64)	0.57 (-0.9, 2.4)
5	3/ 2	**12.76 (11.0, 15.2)**	0.00 (-0.4, 0.5)	**13.11 (11.4, 15.4)**	**-0.00** (-0.8, 0.5)	**-22.52 (-27.2, -19.2)**	**-3.37 (-4.3, -2.4)**
6	1/ 1	**2.06 (1.1, 3.3)**	-0.49 (-2.4, 0.3)	-0.00 (-0.9, 0.4)	0.07 (-0.1, 1.3)	-0.00 (-1.0, 0.4)	-0.33 (-2.2, 0.2)
7	0/ 1	0.00 (-0.04, 0.02)	0.00 (-0.02, 0.04)	0.00 (-0.03, 0.03)	0.00 (-0.03, 0.03)	0.00 (-0.02, 0.03)	0.00 (-0.03, 0.03)

[a] The columns with "#" indicate the number of participants in each condition and in each group.
[b] The bold letters indicate coefficients significantly different from zero.
[c] The numbers in parentheses indicate 95% credible intervals of the posterior distribution of coefficients for the first and second order polynomial.

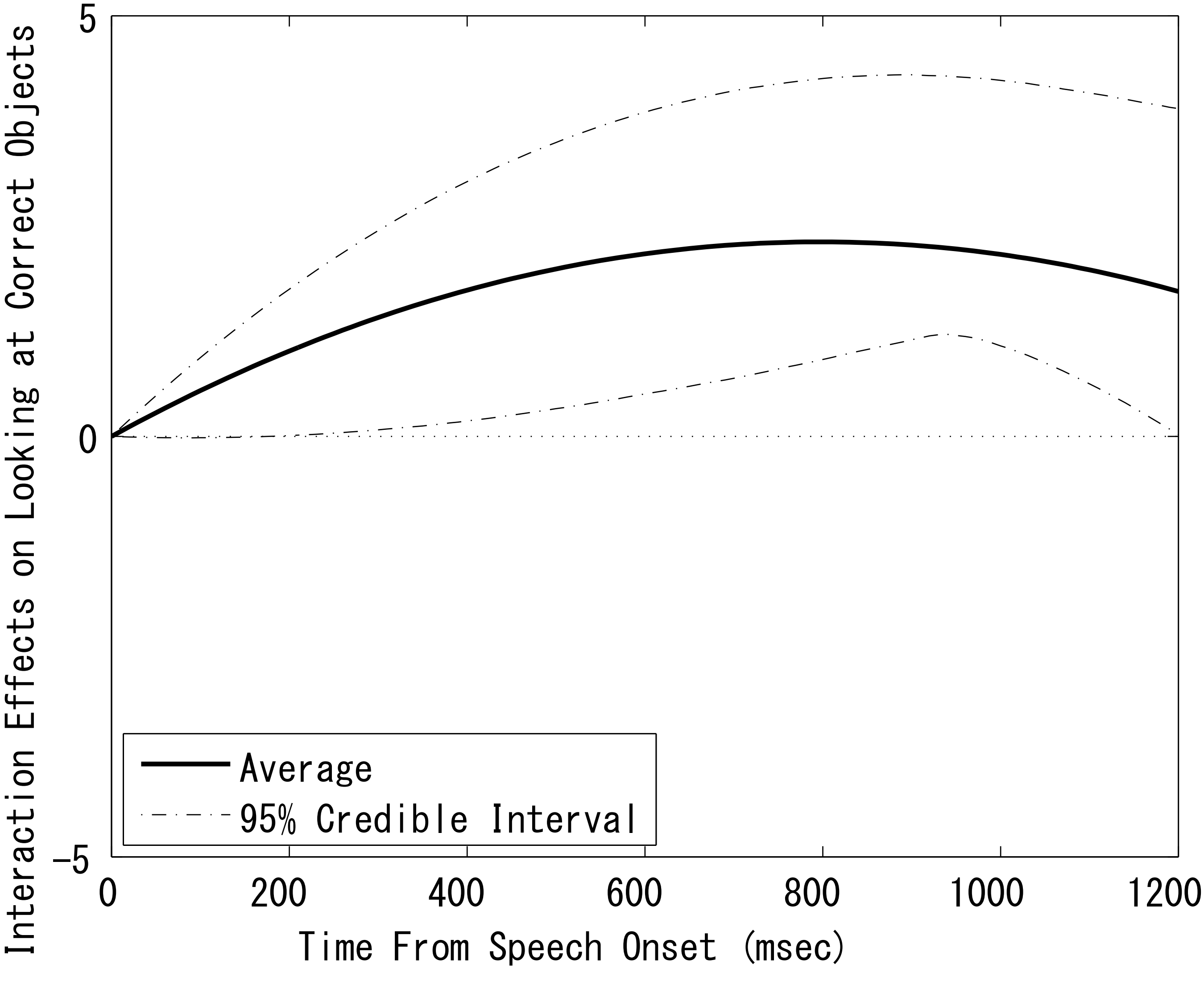

Figure S 1. Effect of the sound symbolism × training interaction term from the Bayesian model, plotted as a function of time, calculated for all infants.
